# Examining cognitive change in magnetic resonance-guided focused ultrasound capsulotomy for psychiatric illness

**DOI:** 10.1038/s41398-020-01072-1

**Published:** 2020-11-11

**Authors:** Benjamin Davidson, Clement Hamani, Ying Meng, Anusha Baskaran, Sachie Sharma, Agessandro Abrahao, Margaret Anne Richter, Anthony Levitt, Peter Giacobbe, Nir Lipsman, Jennifer S. Rabin

**Affiliations:** 1 Division of Neurosurgery, Sunnybrook Health Sciences Centre, University of Toronto, Toronto, ON Canada; 2 Harquail Centre for Neuromodulation, Hurvitz Brain Sciences Program, Sunnybrook Research Institute, Toronto, ON Canada; 3 Sunnybrook Research Institute, Toronto, ON Canada; 4 Department of Medicine (Neurology), University of Toronto, Toronto, ON Canada; 5 Frederick W. Thompson Anxiety Disorders Centre, Sunnybrook Health Sciences Centre, University of Toronto, Toronto, ON Canada; 6 Department of Psychiatry, Sunnybrook Health Sciences Centre, Toronto, Canada; 7 Rehabilitation Sciences Institute, University of Toronto, Toronto, ON Canada

**Keywords:** Neuroscience, Depression

## Abstract

Magnetic resonance-guided focused ultrasound (MRgFUS) anterior capsulotomy is a novel treatment option for patients with refractory obsessive compulsive disorder (OCD) or major depressive disorder (MDD). However, there is concern that lesional psychiatric surgery procedures may have adverse effects on cognition. In this study, we examined whether MRgFUS capsulotomy causes cognitive decline in patients with psychiatric illness. Ten patients with refractory OCD (*n* = 5) or MDD (*n* = 5) underwent MRgFUS capsulotomy. Cognitive functioning was measured at baseline as well as 6 months and 12 months postoperatively, with a battery of neuropsychological tests assessing domains of executive function, memory, and processing speed. Scores were analyzed at the individual-level, and changes ≥2 standard deviations were considered clinically significant. We also examined whether changes in clinical symptoms were associated with changes in cognitive performance. At baseline intellectual functioning was in the average to high-average range for the group. Following MRgFUS capsulotomy, there were no deteriorations in cognition that reached ≥2 standard deviations at 6 or 12 months. Eight out of ten patients demonstrated a ≥2 standard deviation improvement in at least one cognitive score at 6 or 12 months postoperatively. Improvements in clinical symptoms correlated significantly with self-reported improvements in frontal lobe function (*p* < 0.05), but not with objective measures of cognitive functioning. To summarize, MRgFUS capsulotomy did not result in cognitive decline in this cohort of patients with refractory OCD or MDD, suggesting that this procedure can be offered to patients with a very low risk of cognitive side effects.

## Introduction

Mental health disorders affect 1 in 4 people worldwide, with major depressive disorder (MDD) and obsessive compulsive disorder (OCD) being among the most common^1,2^. Despite repeated trials of guideline-concordant pharmacotherapy and psychotherapy, as many as one-third of patients with MDD or OCD are treatment resistant, emphasizing the need for novel therapies^3,4^. In the most severe cases of treatment-resistant MDD and OCD, psychiatric surgery can be a viable treatment option^5^. Psychiatric surgery refers to neurosurgical procedures such as deep brain stimulation (DBS) or lesional surgery, whereby a critical node in the limbic circuitry is stimulated or ablated, respectively^6^.

DBS has several appealing features, such as reversibility and titratability, however it requires an open surgical procedure, with intracranial access and attendant surgical risk, as well as permanent implantation of the stimulating electrodes and pulse generator. In addition, despite promising early reports, recent trials have been inconclusive in terms of the benefit^7–11^. Rapid advances in imaging and surgical technology has led to a revival in lesional procedures^12^. Unlike DBS, lesional procedures do not require a permanent implant, sparing patients the device-programming visits and battery-replacement surgeries. Since the advent of stereotactic radiosurgery (SRS) and magnetic resonance-guided focused ultrasound (MRgFUS), lesional procedures can be performed without requiring a skin incision, leading to a reduced incidence of adverse events, such as hemorrhage or infection^6,13,14^.

Among the most commonly performed lesional procedures is the anterior capsulotomy (AC)^13,15^. AC targets the anterior limb of the internal capsule (ALIC), a dense white matter region where fibers from the limbic prefrontal cortex converge towards the ventral striatum and the thalamus^16,17^. Dysfunctional connectivity patterns within this network have been implicated in the pathogenesis of many psychiatric illnesses, including OCD and MDD^17,18^. Lesioning the ALIC is intended to disrupt the aberrant activity within this network, and mitigate clinical symptoms^19^.

AC is most commonly performed in patients with treatment-resistant OCD or MDD, and most case series report significant clinical improvement in ~50% of patients^15,20^. Since the first AC performed by Talairach in 1949, this procedure has undergone refinements and today is performed in one of three ways: radiofrequency ablation (RF), SRS, or MRgFUS^15,21^. RF ablation involves a skin incision, burr hole, and the insertion of an insulated electrode with an exposed tip. This is then used to heat and ablate the target. SRS uses a helmet lined with a synthetic radioactive isotope of cobalt to deliver targeted radiation to the ALIC^22^. Most recently, MRgFUS has been used to create lesions using ultrasonic waves, sparing patients both an incision and harmful ionizing radiation^23^.

Concerns about the potential cognitive effects of AC have limited its more widespread use in clinical practice^24–26^. These concerns may originate, in part, from the widespread mis-use and abuse of psychiatric surgery in the 1950s^27,28^. Despite the significant advances in the field (e.g., the advent of stereotaxy, MRI-guided targeting, and more strict ethical oversight), lesional procedures are still viewed as risky or dangerous by a substantial proportion of psychiatrists^29^ and the general public^30^. Although numerous recent RF and SRS trials have been effective at improving psychiatric symptoms, several of these studies reported persistent postoperative difficulties on cognitive tasks, particularly on tests of executive function^31–35^. The mechanism(s) responsible for the cognitive difficulties following AC are not fully understood, although there appears to be a higher incidence of adverse cognitive events in patients who receive high levels of radiation (during SRS) or undergo retreatment (with RF), both of which result in relatively large lesion volumes^34^. While larger lesion volumes provide a more complete interruption of the dysfunctional neurocircuitry underlying OCD or MDD symptoms, they may be more likely to impact executive abilities, which are subserved by an overlapping set of regions^16,36^.

Given that MRgFUS targeting the ALIC (MRgFUS-AC) generates lesions under real-time MRI and thermographic guidance, and does not involve open surgery or ionizing radiation, it offers the ability to perform more precise limbic lesions, possibly conferring a reduced risk of cognitive impairment when compared with RF and SRS^12^. A group based out of Seoul reported the first series of MRgFUS-AC, where they treated 11 patients with refractory OCD^37^. At 12 and 24 months postoperatively, 6/11 patients were considered clinical responders, and when analyzed at the group level, no deteriorations in cognitive function were observed.

Recently, our group reported preliminary results of two phase-1 trials treating patients with refractory OCD or MDD with MRgFUS-AC^38^. In that report, neuropsychological testing was available on 9/12 patients, and we found stable cognitive performance at the group level following AC. Here, we present a more detailed account of the cognitive safety profile following MRgFUS-AC in a cohort of 10 patients, which includes the 9 patients reported in our previous study, as well as an additional patient who recently completed the trial. In the present study, we extend our previous report to address two main questions: (1) does MRgFUS-AC result in cognitive changes at the individual patient level? and (2) are changes in clinical symptoms associated with changes in cognitive performance? We hypothesized that MRgFUS-AC would result in minimal neurocognitive deterioration, and that any improvements in clinical symptoms, would correlate with cognitive function.

## Methods

### Participants

Ten patients with treatment-resistant OCD or MDD were enrolled in a clinical trial (NCT03421574; NCT03156335) at the Harquail Centre for Neuromodulation (Sunnybrook Health Sciences Centre, Toronto, Canada) for MRgFUS-AC. Patients were referred from clinics within Sunnybrook Health Sciences Centre or from the community. Each patient was assessed by two psychiatrists, as well as the neurosurgical team, to assess eligibility. Detailed inclusion/exclusion criteria were reported in a previous publication^38^. Briefly, patients were required to be refractory to at least three guideline-concordant trials of antidepressants, two trials of augmentation or combination, and at least one trial of psychotherapy^39^. Patients were not restricted from undergoing postoperative medication changes at the discretion of their primary psychiatrist; medication changes are listed in Supplementary Table 1. Informed consent was provided by all subjects.

### MRgFUS procedure

The MRgFUS-AC procedure has been previously described in detail^40^. Briefly, patients underwent a complete head-shave and stereotactic headframe application, followed by bilateral MRgFUS-AC. The ALIC was targeted at ~7 mm anterior to the anterior commissure, and 12 mm lateral to the midline, based on preoperative high-resolution T2 axial and coronal images, as well as intraoperative T2 images. In most patients, the ALIC can be readily visualized and targeted directly with the intraoperative T2 scan. After three low-intensity localizing sonications, a series of high-powered sonications were conducted, in order to produce bilateral lesions of ~7 mm diameter. Following the 3-4 h procedure, the headframe was removed, and patients spent one night in the hospital for observation. On postoperative day 1, patients were discharged home following an MRI to characterize the lesion size and location, and to rule out any adverse radiographic events. Given the nearly instant mechanism of coagulative necrosis by which lesions are generated by MRgFUS, an MRI obtained within 24 h is well suited to capture the full extent of the lesion^41,42^. Supplementary Fig. 1 depicts a representative example of an MRgFUS-AC lesion and its evolution over time.

### Clinical and neuropsychological assessment

Symptom scales and neuropsychological tests were administered by a trained psychometrist. In the group of patients with refractory OCD, clinical symptoms were assessed using the Yale-Brown Obsessive Compulsive Scale (Y-BOCS)^43^. The Y-BOCS is a widely used clinician-administered instrument that assesses obsessive–compulsive symptomatology. Depressive symptoms were assessed in both the OCD and MDD cohorts using the 17-item Hamilton Depression Rating Scale (HAM-D)^44^. The HAM-D is a clinician-administered scale that assess the severity of depressive symptoms.

To evaluate postoperative changes in cognition, a battery of neuropsychological tests was administered at baseline (within 30 days of treatment), and at 6 and 12 months after treatment. The battery assessed baseline intellectual function, as well as changes in a broad range of cognitive domains, including executive function, episodic memory, and processing speed. The battery included the following tests: Wechsler Test of Adult Reading (WTAR)^45^, California Verbal Learning Test-Second Edition (CVLT)^46^, Brief Visuospatial Memory Test-Revised (BVMT-R)^47^, Delis–Kaplan Executive Function System (D-KEFS) Sorting Test^48^, Symbol Digit Modalities Test (oral version)^49^, Iowa Gambling Task (IGT)^50^, and the Frontal Systems Behavior Scale (FrSBe) (self-report version)^51^. Alternate neuropsychological test versions were used where possible to minimize practice effects. Specifically, we alternated between two versions of the CVLT and the D-KEFS Sorting Test, and used a different version of the BVMT-R at each of the three testing sessions.

### Statistical analysis

Because alternate versions were used for some cognitive tests, all test scores were standardized using published normative data. We first evaluated group level changes in cognitive function from baseline to 6 and 12 months postoperatively using a series of Wilcoxon signed rank tests. Next, we examined changes in cognitive performance at the individual patient level. Each patient’s 6- and 12-month scores were compared with their baseline scores. To identify changes beyond those attributable to practice effects or random error, only changes ≥2 standard deviations (SDs) were considered clinically relevant. Finally, we examined whether changes in clinical symptoms were associated with changes in cognitive performance. To do so, we performed Spearman correlations between percent change scores from baseline to 12 months for the clinical scales (HAM-D for patients with MDD, or Y-BOCS for patients with OCD) with percent change scores for each cognitive measure over the same time period. All statistical analyses were performed in R (Version 3.6.0).

## Results

### Clinical response

There were no serious adverse radiographic or clinical events. Clinical results are displayed in Table 1. At 6 months postoperatively, 1/5 patients with OCD and 0/5 patients with MDD met pre-established responder status (as defined by ≥35% improvement from baseline on the Y-BOCS for patients with OCD patients and ≥50% improvement from baseline on the HAM-D for patients with MDD). At 12 months postoperatively, 3/5 patients with OCD and 1/5 patients with MDD met responder status. The former included the one patient with OCD who met responder status at 6 months—this patient continued to show improvement at 12 months (Patient 1). In three of the four MDD patients who were classified as non-responders, their HAM-D scores worsened by 9–43% at 12 months compared to baseline (Patients 7, 8, 10). The score of the remaining patient with MDD remained stable (Patient 9).

**Table 1:** Clinical outcomes.

Subject	Diagnosis	Y-BOCS				HAM-D			
		Baseline	6 Months	12 Months	% Change	Baseline	6 Months	12 Months	% Change
1	OCD	39	21^a^	18^a^	−54	25	12	9	−64
2	OCD	30	27	19^a^	−37	8	10	7	−12.5
3	OCD	39	29	20^a^	−49	22	18	16	−27
4	OCD	29	23	23	−21	21	23	16	−24
5	OCD	40	39	36	−10	28	28	26	−7
6	MDD	–	–	–		25	19	3^a^	−88
7	MDD	–	–	–		23	19	25	+9
8	MDD	–	–	–		21	26	28	+33
9	MDD	–	–	–		25	26	25	0
10	MDD	–	–	–		21	27	30	+43

*OCD* obsessive compulsvie disorder, *MDD* major depressive disorder, *Y-BOCS* Yale-Brown Obsessive Compulsive Scale, *HAM-D* 17-item Hamilton Depression Rating Scale, % Change denotes change from baseline to 12 months, negative scores represent a decrease in symptoms postoperatively, whereas positive scores represent an increase in symptoms postoperatively.

^a^Indicates that the subject met responder status as defined by ≥ 35% improvement on the Y-BOCS for patients with OCD and ≥ 50% improvement on the HAM-D for patients with MDD).

### Neuropsychological testing—preoperative level

Based on the WTAR scores, patients’ baseline intellectual functioning was estimated to be in the average or high-average range. When considering baseline scores on the remaining neuropsychological tests, several patients demonstrated relative weaknesses (i.e., ≥1 SD below patients’ respective performance on the WTAR) on tests assessing processing speed and decision-making. Across all patients, most memory scores fell within expected ranges (average or above), although relative weaknesses were observed on select measures for a few patients (i.e., non-verbal immediate recall on BVMT-R, recognition discrimination on CVLT). All patients had performances within expected ranges on a complex problem-solving task (D-KEFS Sorting Test). On a self-report measure assessing “frontal systems” behaviors, (FrSBe), 4/10 patients endorsed clinically significant symptoms of disinhibition, 9/10 endorsed clinically significant symptoms of apathy, and 7/10 endorsed clinically significant symptoms of executive dysfunction (Supplementary Table 2).

### Neuropsychological testing—postoperative changes

At the group level, postoperative cognitive scores were stable or mildly improved at both 6 and 12 months postoperatively compared to baseline (Table 2). Regarding performance-based tests at 6 months, significant improvements from baseline were observed on the Symbol Digit Modality Test and the D-KEFS Sorting Test (sorting and description scores) (*p* < 0.05). At 12 months, significant improvements from baseline were observed on the BVMT-R (immediate recall and delayed recall scores) and the D-KEFS Sorting Test (sorting and description scores) (*p* < 0.05). Self-report measures also improved following treatment, with patients endorsing fewer symptoms of apathy at 6 and 12 months compared to baseline (FrSBe apathy score), and endorsing fewer overall frontal symptoms (FrSBe total score) at 12 months compared to baseline (*p* < 0.05). All other scores remained stable (*p* > 0.05).

**Table 2:** Mean baseline, 6, and 12-month postoperative neuropsychological scores (*n* = 10 subjects).

	Baseline	6 months	12 months	Decline ≥ 2 SD		Improvement ≥ 2 SD	
				B-12mos	B-6mos	B-12mos	B-6mos
CVLT total recall^a^	53.3 (8.2)	55 (7.0)	52.7 (12.2)	0	0	0	0
CVLT delayed free recall^c^	0.0 (0.8)	−0.1 (1.0)	0.3 (0.8)	0	0	0	0
CVLT delayed cued recall^c^	−0.4 (1.4)	0.2 (0.8)	0.2 (0.8)	0	0	1 (pt8)	1 (pt8)
CVLT delayed recognition discrimination^c^	−0.2 (1.8)	0.1 (0.7)	0.2 (0.8)	0	0	1 (pt8)	1 (pt8)
BVMT-R immediate recall^a^	41.1 (16.1)	47.6 (11.2)	51.7 (13.0)*	0	0	3 (pt1,6,10)	1 (pt10)
BVMT-R delayed recall^a^	44.5 (16.8)	51.1 (9.4)	53.4 (11.4)*	0	0	1 (pt3)	0
Oral SDMT^c^	−1.0 (0.9)	−0.5 (0.9)*	−0.6 (1.0)	0	0	0	0
D-KEFS Sorting Test: correct sorts^b^	9.8 (1.8)	12.5 (1.9)*	13.4 (3.1)*	0	0	4 (pt2,3,6,8)	0
D-KEFS Sorting Test: description score^b^	10.3 (2.5)	12.4 (2.2)*	13.1 (3.3)*	0	0	1 (pt8)	1 (pt8)
IGT total score^a^	45.5 (8.9)	47.0 (9.0)	49.8 (7.0)	0	0	1 (pt4)	0
FrSBe total score^a^	73.9 (14.0)	66.1 (14.7)	63.5 (17.0)*	0	0	2 (pt3,6)	1 (pt1)
FrSBe disinhibition score^a^	57.8 (20.4)	52.6 (17.7)	50.4 (18.6)	0	0	3 (pt3,6)	2 (pt3,6)
FrSBe apathy score^a^	88.9 (17.8)	79.6 (18.2)*	76.4 (25.3)*	0	0	2 (pt3,6)	1 (pt1)
FrSBe dysexecutive score^a^	68.9 (14.1)	62.7 (8.6)	60.6 (12.6)	0	0	2 (pt3,6)	1 (pt1)

Scores were standardized with published normative data, and reported in the following format: ^a^ T-Score (mean of 50, SD of 10); ^b^ Scaled Score (mean of 10, SD of 3); ^c^ *Z*-Score (mean of 0; SD of 1).

*B-12mos* Pt change from baseline to 12 months, *B-6mos* change from baseline to 6 months, *Pt* patient, *CVLT* California Verbal Learning Test, *BVMT-R* Brief Visuospatial Memory Test-Revised, *SDMT* Symbol Digit Modalities Test, *FrSBe* Frontal Systems Behavior Scale—self-version (lower scores represent fewer behavioral symptoms), *IGT* Iowa Gambling Task.

*Denotes *p* < 0.05 on a Wilcoxon signed rank test in comparison to baseline.

Note that 7/10 patients completed the IGT at 6 months, and 9/10 patients completed the ψ IGT at 12 months.

**Fig. 1:**
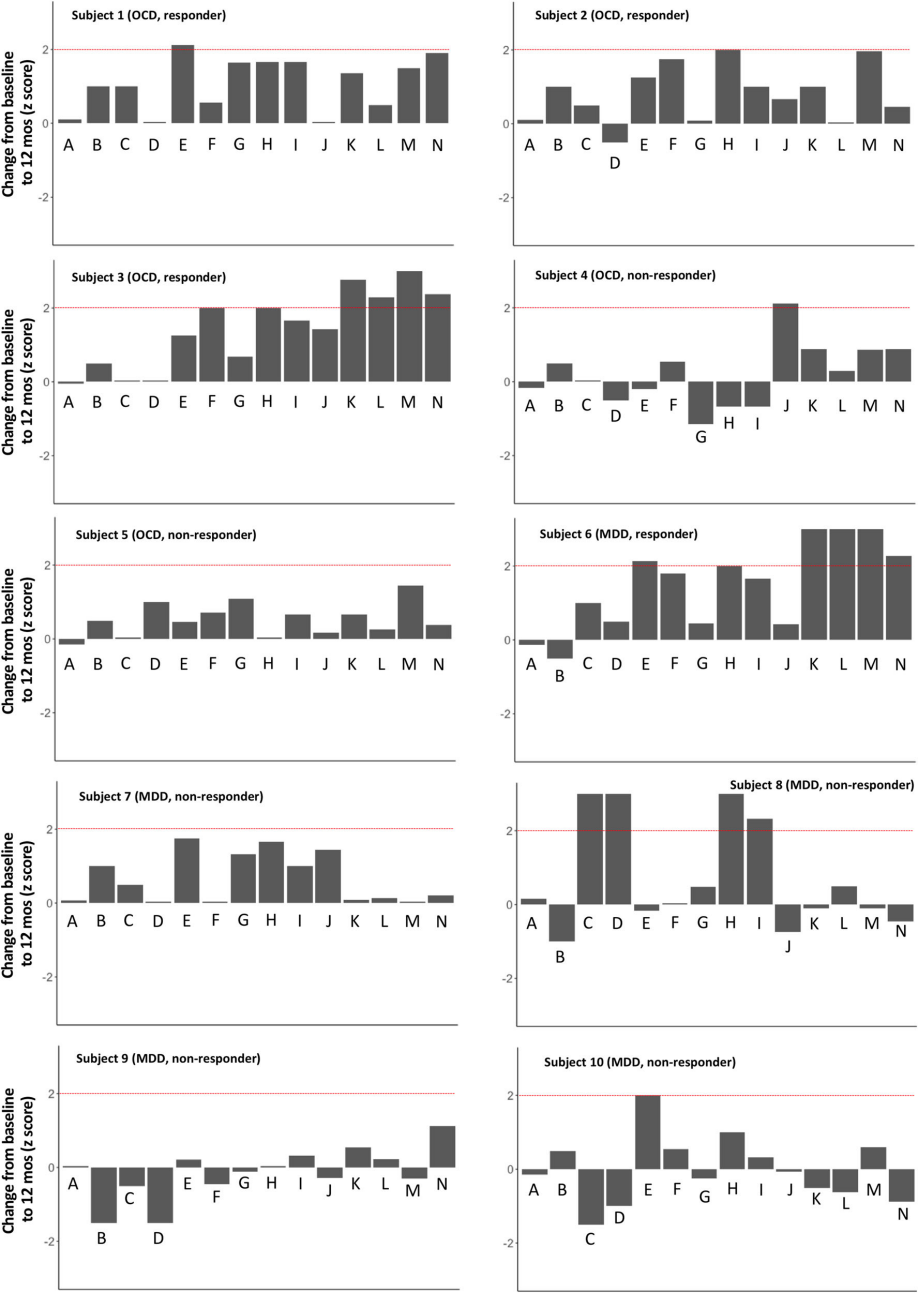
Individual barplots representing change on neuropsychological tests following MRgFUS-AC. The *y*-axis represents change from baseline to 12 months for each patient as a standardized score (*z* score). Deviations below 0 represent decline and deviations above 0 represent improvement. The dotted red lines indicate a clinically significant improvement (i.e., 2 SD improvement). Since no patients’ performance declined ≥2 SD, no corresponding line representing a decline was added). Subject 1 did not complete the Iowa Gambling Task (IGT) at 12-months and therefore the subject’s 6-month scorewas used to compute a change score. A = CVLT total recall; B = CVLT delayed free recall; C = CVLT delayed cued recall; D = CVLT delayed recognition discrimination; E = BVMT-R immediate recall; F = BVMT-R delayed recall; G = Symbol Digit Modalities Test; H = D-KEFS Sorting Test sorting score; I = D-KEFS Sorting Test description score; J = IGT; K = FrSBe total score; L = FrSBe disinhibition score; M = FrSBe apathy score; N = FrSBe dysexecutive score.

At the individual level, we did not observe a decline on any neuropsychological test that was ≥2 SD from baseline (at 6 or 12 months postoperatively) (Fig. 1, Table 2, Supplementary Table 2). Even at a more liberal threshold of 1.5 SD, across all patients very few scores declined. Specifically, at 6 months, there was only 1 patient with 1 score that declined and at 12 months three scores from three different patients declined in this range. In terms of improvements, four patients showed improved performance ≥2 SD on at least one score at 6 months, and seven patients showed ≥2 SD improvement on at least one measure at 12 months. There was heterogeneity with respect to the test scores that showed improvement across patients. Notably, the two patients with the greatest number of test score improvements in this range were both responders (OCD patient #3 and MDD patient #6).

### Correlation between neuropsychological testing and clinical outcome

**Fig. 2:**
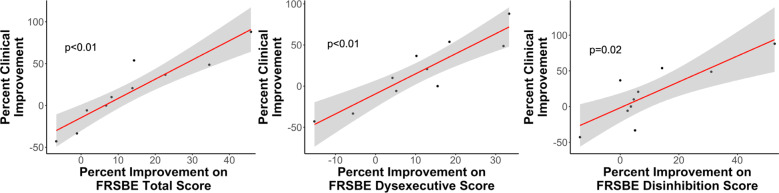
Correlations between the FrSBe subscores and clinical scores. The scatterplots display the relationship between the percentage improvement on the FrSBe-total, FrSBe-dysexecutive scores, and FrSBe-disinhibition scores, with the percentage symptom improvement at 12 months postoperatively (as measured by the Y-BOCS for patients with OCD or the HAM-D for patients with MDD). No other neuropsychological measure significantly correlated with clinical symptoms at 12 months postoperatively.

Finally, we examined whether changes in clinical symptoms were associated with changes in cognitive performance from baseline to 12 months postoperatively. We found that percent improvement on clinical scales (Y-BOCS for the patients with OCD, and HAM-D for the patients with MDD) correlated significantly with improvements on self-report measures assessing frontal-executive abilities, specifically, the FrSBe total score, the FrSBe dysexecutive score, and the FrSBe disinhibition score (*p* < 0.05) (Fig. 2, Supplementary Table 3).

## Discussion

In 10 patients with refractory OCD or MDD who underwent MRgFUS-AC, neuropsychological test scores were generally stable or mildly improved postoperatively at 6 and 12 months. This pattern of performance was observed on tests of processing speed, memory, executive function, as well as on a self-report measure assessing frontal executive abilities. In addition, we found that improvements in clinical symptoms (i.e., obsessive-compulsive or depressive symptoms) correlated significantly with improvements on self-report measures of apathy and disinhibition, but not with performance-based tasks. Together, these findings suggest that MRgFUS-AC is a safe treatment option for patients with refractory OCD or MDD and when effective may have secondary benefits in terms of improving frontal executive abilities.

Previous RF and SRS capsulotomy studies have reported postoperative decline on tests of executive function, when measured ≥6 months from treatment^31–35^. By contrast, and consistent with the only other published MRgFUS-AC study, we found that patients’ postoperative test scores remained stable or mildly improved compared to baseline scores. This pattern of performance was observed on tests of executive function as well as on tests of memory and processing speed. The improved cognitive safety profile of MRgFUS-AC relative to RF and SRS may be because the lesions produced in this trial were smaller than those typically produced by RF and SRS^34,37,42,52,53^ and that none of our patients underwent multiple surgical treatments, which was the case in many of the previously reported RF and SRS series^34,35^.

**Fig. 3:**
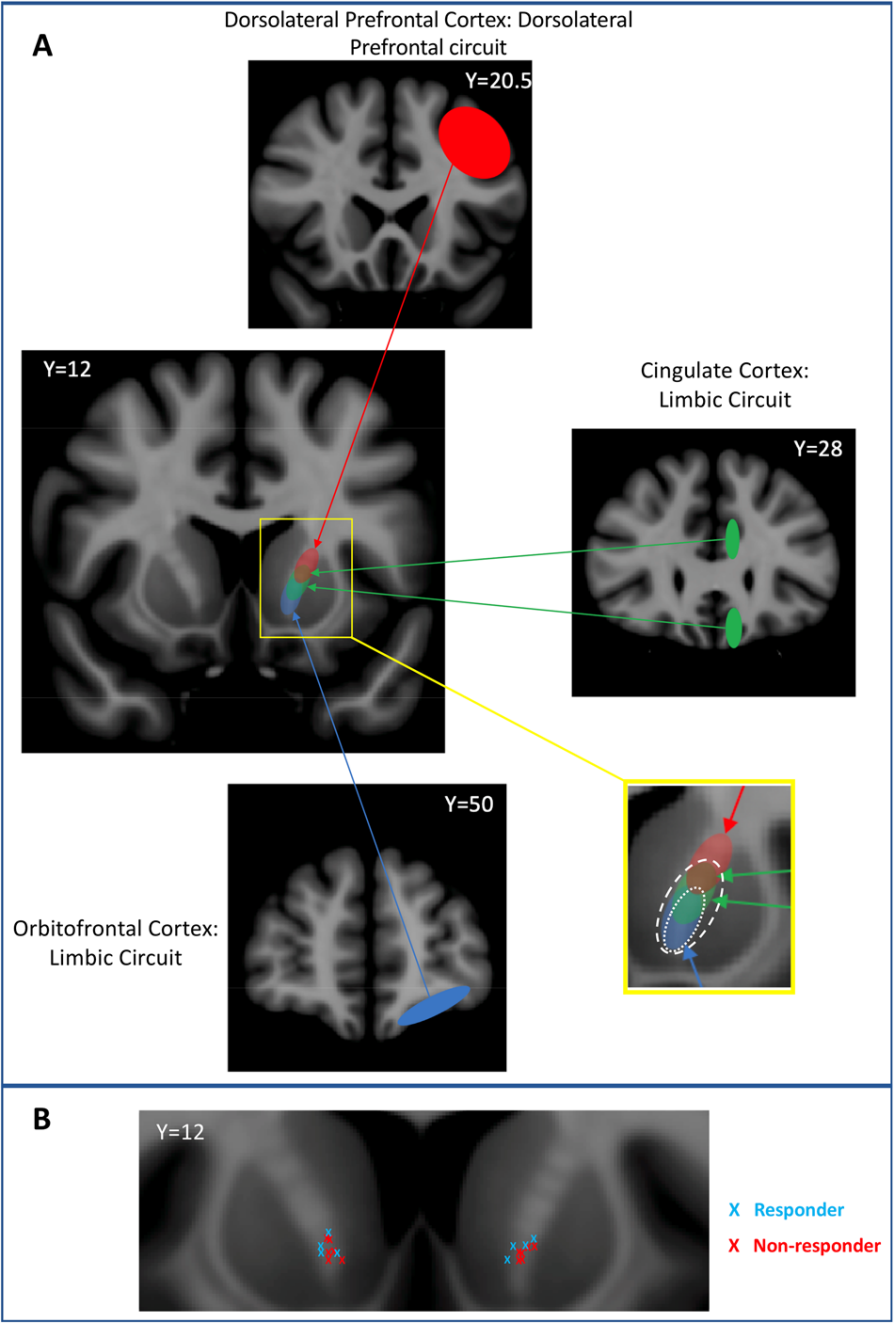
MRgFUS targeting of the ventral ALIC. **A** Schematic displaying the overlap of executive and limbic circuit axonal fibers in the ALIC. Fibers projecting from the orbitofrontal cortex (blue) and cingulate cortex (green) are found more ventrally within the ALIC, and are associated with the limbic circuit. Fibers projecting from the dorsolateral prefrontal cortex (red), are found more dorsally in the ALIC, and are associated with the executive circuit. As demonstrated in the yellow inset box, larger lesions (lesions indicated by dotted white lines) interrupt a greater proportion of limbic fibers, but also impinge on the executive circuit. *Y* coordinates indicate the anterior–posterior position of each slice in Montreal Neurological Institute (MNI) space. Regions are drawn for illustrative purposes and are not to scale. **b** Coronal representation of lesion centroids in MNI-space, coded for clinical responders (light blue), and non-responders (red).

Larger lesions are more likely to result in cognitive impairment, because the abnormally functioning circuits implicated in OCD and MDD overlap with the circuit thatsupports executive abilities^54–56^. Executive function is supported by a circuit involving the dorsolateral prefrontal cortex and, its projections to the central striatum, whereas obsessive-compulsive and depressive symptoms have been linked to a limbic circuit, involving the cingulate and orbitofrontal cortices, which project to the ventromedial striatum (including the nucleus accumbens). Within the ALIC, these two circuits are anatomically separated by a ventromedial–dorsolateral gradient^57,58^. By targeting the ventromedial aspect of the ALIC during AC, the goal is to disrupt limbic fibers, however, some degree of disruption to the dorsolateral prefrontal circuit supporting executive function may occur, especially with larger lesions (see Fig. 3 for an anatomical depiction of this phenomenon). There is likely a volume threshold beyond which further expansion of a lesion will result in worsening cognitive impairment. There is also likely a minimum lesion volume necessary to produce a clinical benefit. This balancing act is analogous to performing thalamotomies for essential tremor, where a lesion volume >170 mm^3^ results in a greater incidence of adverse motor outcomes, but for lesions <170 mm^3^, the location the location is critical to mitigate the tremor^59,60^. Thus, a lesion must be large enough to result in a clinically beneficial disruption of the limbic circuitry but small enough to avoid cognitive sequelae.

Given that we did not observe a decline in cognition in our cohort, it is not possible to comment on an upper safe limit for lesion volume. It would be interesting for future MRgFUS-AC studies to investigate slightly larger lesion volumes in an attempt to improve responder rates, however this may result in a greater rate of postoperative cognitive impairment, particularly in executive abilities. With respect to SRS, there is strong evidence to suggest that two contiguously stacked lesions may offer a better clinical response than a single lesion; this approach has yet to be studied with MRgFUS^53^. To add further complexity to the issue of a safe upper limit of a lesion volume, there is substantial heterogeneity in the functional organization of the striatum across individuals^58^, suggesting that identical lesions may have differing effects across subjects.

Our findings are consistent with the results from psychiatric DBS studies, which consistently report that DBS is safe from a cognitive perspective^7,8,61,62^. The safety profile of DBS likely stems from two main factors. First, the volume of tissue activated (VTA) surrounding the DBS contact is smaller than that of a typical RF lesion. Second, DBS allows for titration, and therefore, should cognition be impacted, stimulation parameters can be changed to quickly resolve the issue. Based on the cognitive findings reported here, MRgFUS-AC could be considered as a reasonable alternative to DBS for patients with OCD or MDD, though this will also depend on patient/physician preference, resource availability, and logistical considerations.

The second question we addressed in this study focused on whether changes in clinical symptoms are associated with changes in cognitive performance. We found that improvements in clinical symptoms (obsessive-compulsive or depressive symptoms) correlated significantly with improvements on self-reported measures of frontal-executive abilities, including apathy and disinhibition. By contrast, we did not observe associations with any of the performance-based cognitive measures, including tests of executive function. This is not unexpected given that performance on objective tests of executive function do not always correlate with self-report measures^63^. The observed correlation suggests that reducing obsessive-compulsive or depressive symptoms may be accompanied by improvements in frontal functions. It may be that the amelioration of obsessive-compulsive or depressive symptoms leads to secondary improvements in frontal-executive abilities or that the successful interruption of abnormally functioning frontal-striatal circuits results in downstream improvement to both obsessive-compulsive or depressive symptoms and frontal-executive abilities. An alternative possibility is that improvement in patients’ obsessive-compulsive or depressive symptoms positively influenced their perception of their frontal-executive abilities, which were then rated more favorably.

The present study has several important limitations. First, the sample size was relatively small (*n* = 10) and we did not include a control group. Additional studies with larger sample sizes and control groups are required to obtain a more comprehensive understanding of the impact of MRgFUS-AC on cognitive functioning. Further, although we attempted to minimize practice effects by using alternate versions of tests where possible, we cannot rule out the possibility that repeat testing impacted the results. In addition, only 4 of the 10 patients in our cohort met responder status, which limited our ability to assess correlations with clinical improvement.

## Conclusion

Concerns regarding postoperative decline in cognitive function have historically diminished the use of surgical interventions to treat refractory psychiatric disease. Here, we show that MRgFUS-AC does not adversely impact cognitive function across several domains, suggesting that it is a safe treatment option for individuals with treatment-resistant OCD or MDD. In addition, we observed that improvement in obsessive-compulsive or depressive symptoms following MRgFUS-AC may be accompanied by improvement in self-reported frontal-executive abilities. Referring physicians and patients seeking surgical interventions should be reassured by this favorable cognitive side effect profile.

## Supplementary information

Supplementary Material
