# Supplementary material for: Examining cognitive change in magnetic resonance-guided focused ultrasound capsulotomy for psychiatric illness
Source: Transl Psychiatry. 2020 Nov 11;10:397. doi: 10.1038/s41398-020-01072-1 (PMC7658970; doi:10.1038/s41398-020-01072-1)
Supplement: Supplementary file 1 — Supplementary Material [file 41398_2020_1072_MOESM1_ESM.docx]

**Supplementary Figure 1.** Evolution of MRgFUS-Capsulotomy lesions

Figure taken with permission from: Davidson et al. *Molecular Psychiatry*. 2020 (Supplementary Material)

**Supplementary Table 1. Medication Changes**

| Subject | Diagnosis | Psychiatric medications at time of MRgFUS | Medication changes during follow-up period | |
| --- | --- | --- | --- | --- |
| 1 | OCD | Amitriptyline 150mg BID, citalopram 40mg OD, risperidone 1.5mg OD | | - |
| 2 | OCD | Sertraline 250mg OD, risperidone 2mg OD, lorazepam 0.5mg BID, clonazepam 0.5 md TID, | | - |
| 3 | OCD | Paroxetine 40 mg OD, clonazepam 0.5mg BID, memantime 10 mg OD | | Added trazodone 75 mg OD PRN for sleep |
| 4 | OCD | Clomipramine 300mg OD, desvenlafaxine 200mg OD, lurasidone 40 mg OD, lorazepam 1mg OD | | Stopped lurasidone,  tried on risperidone but that was stopped,  Lurasidone replaced with risperidone  Clomipramine decreased to 50mg OD |
| 5 | OCD | Nil^τ^ | | Trial of lurasidone 20 mg OD, stopped due to lack of efficacy |
| 7 | MDD | Risperidone 4.5mg daily (divided), ketamine 0.01mg twice weekly, desipramine 40mg OD, clonazepam 1.75 mg OD. | | Decreased risperidone to 3.0mg daily (divided)  Added clonazepam 0.25mg BID PRN,  Decreased desipramine to 20mg OD,  Added vilazadone 2.5mg OD |
| 8 | MDD | Quetiapine 50mg OD, buproprion 150mg OD, ketamine nasal spray 150mg weekly, gabapentin 2000 daily (divided), trazodone 50mg OD | | - |
| 9 | MDD | Nil^τ^ | | Added chlorpromazine 12.5mg every other day |
| 10 | MDD | Levomilnacapran 120mg OD, asenapine 5mg OD, clonazepam 1mg OD | | Increased levomilnacapran to 140mg OD |
| 12 | MDD | Levothyroxine 0.025mg OD^τ^ | | Started fluoxetine, 40mg OD, but discontinued due to lack of effect |

^τ^All psychiatric medications had been stopped due to lack of effect; OD: once daily; BID: twice daily;

TID: three times daily; PRN: as needed

**Supplementary Table 2.** Standardized Neuropsychological Test Scores for Individual Patients

|  | OCD | | | | | MDD | | | | |
| --- | --- | --- | --- | --- | --- | --- | --- | --- | --- | --- |
|  | Pt 1 | Pt 2 | Pt 3 | Pt 4 | Pt 5 | Pt 6 | Pt 7 | Pt 8 | Pt 9 | Pt 10 |
| **WTAR**^a^ |  |  |  |  |  |  |  |  |  |  |
| Baseline | 106 | 97 | 98 | 108 | 108 | 119 | 114 | 112 | 117 | 114 |
| **CVLT-II Total recall**^b^ |  |  |  |  |  |  |  |  |  |  |
| Baseline | 56 | 47 | 54 | 65 | 40 | 44 | 60 | 63 | 55 | 49 |
| 6mos | 62 | 45 | 49 | 65 | 47 | 51 | 56 | 60 | 62 | 53 |
| 12mos | 62 | 52 | 51 | 54 | 34 | 38 | 64 | 73 | 57 | 42 |
| **CVLT-II Delayed free recall**^c^ |  |  |  |  |  |  |  |  |  |  |
| Baseline  6mos  12mos | 0.5  1  1.5 | 0 | 0.5 | 0.5 | -2 | 0 | 0 | 1.5 | 1 | -0.5 |
|  |  | -1 | -0.5 | -1 | -1 | -1 | 0.5 | 1.5 | 1 | -0.5 |
|  |  | 1 | 1 | 1 | -1.5 | -0.5 | 1 | 0.5 | -0.5 | -1 |
| **CVLT Delayed cued recall**^c^ |  |  |  |  |  |  |  |  |  |  |
| Baseline  6mos  12mos | 0  1  1 | 0.5  -0.5  1 | 0.5  0.5  0.5 | 0.5  0  0.5 | -1  0  -1 | -0.5  -1.5  0.5 | 0  0.5  0.5 | -4  1  0.5 | 0.5  1  0 | 0  0  -1.5 |
| **CVLT Delayed recognition discrimination**^c^ |  |  |  |  |  |  |  |  |  |  |
| Baseline  6mos  12mos | 1  0.5  1 | 0.5  0  0 | 1  0.5  1 | 0  0  -0.5 | -0.5  0.5  0.5 | 0  -0.5  0.5 | 0.5  1  0.5 | -5  0  0.5 | 1  0  -0.5 | -0.5  -1.5  -1.5 |
| **BVMT-R Immediate** **recall**^b^ |  |  |  |  |  |  |  |  |  |  |
| Baseline  6mos  12mos | 39  52  60 | 48 | 20 | 56 | 24 | 20 | 46 | 67 | 54 | 37 |
|  |  | 46 | 36 | 56 | 39 | 28 | 63 | 57 | 42 | 57 |
|  |  | 60 | 32 | 54 | 29 | 41 | 63 | 65 | 56 | 57 |
| **BVMT-R Delayed** **recall**^b^ |  |  |  |  |  |  |  |  |  |  |
| Baseline  6mos  12mos | 55  55  61 | 46  46  63 | 22  41  42 | 51  51  57 | 24  43  31 | 23  36  41 | 63  63  63 | 64  64  64 | 54  42  56 | 43  62  49 |
| **Oral SDMT**^c^ |  |  |  |  |  |  |  |  |  |  |
| Baseline  6mos  12mos | -2.1  -1.2  -0.5 | -1.2 | -1.7 | -0.4 | -1.4 | -0.7 | -1.2 | 1.3 | -1.4 | -1.0 |
|  |  | -0.9 | -1.3 | -0.5 | -0.9 | -0.6 | -0.4 | 1.8 | -1.3 | 0.1 |
|  |  | -1.1 | -1.0 | -1.6 | -0.3 | -0.3 | 0.1 | 1.8 | -1.5 | -1.3 |
| **D-KEFS Sorting Test: Correct sorts**^d^ |  |  |  |  |  |  |  |  |  |  |
| Baseline  6mos  12mos | 10  12  15 | 9 | 6 | 10 | 9 | 9 | 10 | 11 | 12 | 12 |
|  |  | 11 | 10 | 10 | 12 | 14 | 15 | 14 | 15 | 12 |
|  |  | 15 | 12 | 8 | 9 | 15 | 15 | 18 | 12 | 15 |
| **D-KEFS Sorting Test: Description score**^d^ |  |  |  |  |  |  |  |  |  |  |
| Baseline  6mos  12mos | 10  12  15 | 10 | 7 | 10 | 9 | 10 | 9 | 9 | 16 | 13 |
|  |  | 11 | 9 | 10 | 12 | 13 | 14 | 15 | 16 | 12 |
|  |  | 13 | 12 | 8 | 7 | 15 | 14 | 16 | 17 | 14 |
| **FrSBe: Total score**^b^ |  |  |  |  |  |  |  |  |  |  |
| Baseline  6mos  12mos | 91  54  78 | 44  39  34 | 81  64  53 | 65  61  56 | 73  72  67 | 83  69  45 | 64  62  63 | 89  96  90 | 74  69  69 | 75  75  80 |
| **FrSBe: Disinhibition score**^b^ |  |  |  |  |  |  |  |  |  |  |
| Baseline  6mos  12mos | 35 | 35 | 74 | 49 | 65 | 74 | 41 | 99 | 55 | 51 |
|  | 34 | 35 | 51 | 46 | 71 | 50 | 38 | 92 | 53 | 56 |
|  | 30 | 35 | 51 | 46 | 42 | 35 | 40 | 94 | 53 | 58 |
| **FrSBe**: **Apathy score**^b^ |  |  |  |  |  |  |  |  |  |  |
| Baseline  6mos  12mos | 110  72  106 | 55  42  36 | 88  74  58 | 69  69  60 | 82  73  68 | 97  87  50 | 92  90 92 | 98  105  99 | 84  82  87 | 114  102  108 |
| **FrSBe**: **Dysexecutive score**^b^ |  |  |  |  |  |  |  |  |  |  |
| Baseline  6mos  12mos | 103  59  84 | 49  49  44 | 72  62  49 | 70  64  61 | 71  73  68 | 69  59  46 | 58  56  55 | 67  80  71 | 71  63  60 | 59  62  68 |
| **IGT Total score**^b^ |  |  |  |  |  |  |  |  |  |  |
| Baseline  6mos  12mos | 62  62 | 40  47 | 42  57 | 33  39  54 | 44  45  46 | 33  48 | 39  50  53 | 42  35  35 | 54  53  51 | 56  45  57 |

Scores were standardized with published normative data, and reported in the following format:

^a^ Standard score (mean of 100, SD of 15); ^b^T-Score (mean of 50, SD of 10); ^c^ Z-Score (mean of 0; SD of 1); ^d^Scaled Score (mean of 10, SD of 3).

Pt = patient; CVLT = California Verbal Learning Test; BVMT-R = Brief Visuospatial Memory Test-Revised; SDMT = Symbol Digit Modalities Test; FrSBe = Frontal Systems Behavior Scale – Self version (lower scores represent fewer behavioural symptoms); IGT = Iowa Gambling Task.

**Supplementary Table 3.** Spearman correlations between changes in neuropsychological scores and percent improvement in clinical symptoms at 12 months

|  | Rho | p |
| --- | --- | --- |
| CVLT-II Total recall | 0.02 | 0.97 |
| CVLT-II Delayed free recall | -0.21 | 0.56 |
| CVLT Delayed cued recall | -0.36 | 0.31 |
| CVLT delayed recognition discrimination | -0.17 | 0.63 |
| BVMT-R Immediate recall | -0.43 | 0.22 |
| BVMT-R Delayed recall | -0.55 | 0.10 |
| Oral SDMT | -0.25 | 0.49 |
| D-KEFS Sorting Test: Correct sorts | -0.42 | 0.22 |
| D-KEFS Sorting Test: Description score | -0.05 | 0.89 |
| FrSBe Total score | 0.96 | **<0.01*** |
| FrSBe Disinhibition score | 0.73 | **0.02*** |
| FrSBe Apathy score | 0.65 | 0.05 |
| FrSBe Dysexecutive score | 0.88 | **<0.01*** |
| IGT Total score | -0.28 | 0.43 |

CVLT = California Verbal Learning Test; BVMT-R = Brief Visuospatial Memory Test-Revised; SDMT = Symbol Digit Modalities Test; FrSBe = Frontal Systems Behavior Scale – Self version (lower scores represent fewer behavioural symptoms); IGT = Iowa Gambling Task.
